# Long-Term Lower Respiratory Symptoms among World Trade Center Health Registry Enrollees Following Hurricane Sandy

**DOI:** 10.3390/ijerph192113738

**Published:** 2022-10-22

**Authors:** Sean H. Locke, Lisa M. Gargano, Howard E. Alper, Jennifer Brite

**Affiliations:** 1 World Trade Center Health Registry, Division of Epidemiology, New York City Department of Health and Mental Hygiene, Long Island City, NY 11101, USA; 2 Department of Health & Human Performance, York College of City University of New York (CUNY), New York, NY 11451, USA; 3 CUNY Institute for Demographic Research, City University of New York (CUNY), New York, NY 11451, USA

**Keywords:** 9/11 disaster, lower respiratory symptoms, PTSD, World Trade Center

## Abstract

Several studies showed an association between lower respiratory tract symptoms (LRS) and exposure to the 9/11 terrorist attack. However, few studies have examined the long-term impact of natural disasters on those with prior respiratory distress. The present study aims to assess the impact of Hurricane Sandy on persistent LRS among people exposed to the World Trade Center (WTC) terrorist attack. The analytic sample consisted of WTC Health Registry enrollees who completed survey waves 1, 3, and 4 and the Hurricane Sandy Survey and did not report LRS before the WTC terrorist attack. The log binomial was used to assess the association between the impact of Hurricane Sandy and persistent LRS. Of 3277 enrollees, 1111 (33.9%) reported persistent LRS post-Sandy. Participants of older age, males, lower household income, current smokers, and those with previous asthma were more likely to report persistent LRS. In separate adjusted models, multiple Sandy-related inhalation exposures (relative risk (RR): 1.2, 95% CI: 1.06–1.37), Sandy-related PTSD (RR: 1.27, 95% CI: 1.15–1.4), and Sandy LRS (RR: 1.64, 95% CI: 1.48–1.81) were associated with persistent LRS post-Sandy. Our findings suggest that respiratory protection is important for everyone performing reconstruction and clean-up work after a natural disaster, particularly among those with previous respiratory exposures.

## 1. Introduction

Lower respiratory symptoms (LRS) were one of the most common conditions reported following disaster-related environmental hazards such as the World Trade Center (WTC) attacks on 11 September 2001 [1], Hurricane Sandy in 2012, and the Japanese tsunami of 2011 [2,3]. A previous study found that the prevalence of LRS among both recovery workers and residents was 19% five years after the WTC terrorist attacks [4]. Many of those who experienced the events of 9/11 were also exposed to Hurricane Sandy, which hit New York City on 29 October 2012, flooding large areas of lower Manhattan. The flooding created environmental conditions that could increase the risk of LRS, specifically the dust left behind when the floodwater receded, the mold growth in the flooding area, dust mite, and microbial growth. A previous study demonstrated Hurricane Sandy environmental conditions were associated with LRS about a year after Hurricane Sandy [5]. The effect of multiple environmental exposures on persistent LRS is unknown and has not, to our knowledge, been previously examined.

Persistent LRS has been associated with several negative outcomes, such as increased risk of hospitalization [6] and decreased quality of life. For example, a prior study assessed persistent LRS 10 years after the 9/11 disaster among rescue/recovery workers, local area workers, residents, and passers-by in the World Trade Center Health Registry (Registry) and found that persistent LRS was negatively associated with enrollees’ physical health and mental health [7]. Although the etiology and pathophysiological processes associated with LRS are not fully known, a study utilizing impulse oscillometry [8] concluded that peripheral airway dysfunction and post-traumatic stress disorder (PTSD) were independently associated with the persistence of LRS 12–13 years after the attacks.

Despite research exploring the relationship among PTSD, Hurricane Sandy environmental conditions, and LRS, the literature on long-term LRS due to Hurricane Sandy is limited, particularly among those exposed to 9/11. The present study is a continuation of work conducted by Gargano et al. [5] with the following aims: (1) to describe three Hurricane Sandy-related health impacts: inhalation exposure, PTSD, and LRS; (2) to examine the prevalence of persistent LRS three to four years after the hurricane; (3) to assess whether Hurricane Sandy-related exposures are associated with persistent LRS; and, if so, (4) to determine whether the relationship varies by the presence or absence of prior LRS.

## 2. Materials and Methods

### 2.1. Registry Summary

The World Trade Center Health Registry (Registry) was established in 2002 to monitor the physical and mental health of over 71,000 individuals exposed to the 9/11 terrorist attacks. The Registry includes rescue and recovery workers, lower Manhattan residents living south of Canal Street, schoolchildren, building occupants, passers-by, and area workers south of Chamber Street. The Registry conducts surveys to monitor the long-term health impacts of 9/11, beginning with the baseline survey conducted in 2003–2004 and follow-up surveys conducted in 2006–2007 (wave 2), 2011–2012 (wave 3), 2015–2016 (wave 4), and 2020–2021 (wave 5) [9].

Wave 3 data collection ended on 7 March 2012, just months before Hurricane Sandy. The 43,134 enrollees who completed wave 3 and lived in the tri-state metropolitan area constituted the sampling frame for the Hurricane Sandy survey. The survey included all 4435 enrollees who lived in the Sandy inundation zone, defined by the Federal Emergency Management Agency (FEMA) Modeling Task Force; a random sample of 4435 enrollees who were not from the FEMA-defined inundation zone was selected as a comparison group. Beginning on 28 March 2013, the 8870 enrollees were invited via email or mail to complete a survey regarding their physical and mental health and their Sandy-related exposure. A total of 4558 completed surveys were received (2443 from the inundation zone and 2115 from the non-inundation zone) by 7 November 2013. The Registry and Hurricane Sandy studies were approved by the New York City Department of Health and Mental Hygiene’s Institutional Review Board.

The current study included enrollees who completed the Hurricane Sandy survey as well as Registry waves 1, 3, and 4, and had not reported LRS before the WTC terrorist attack, resulting in a final analytic sample of 3277.

### 2.2. Lower Respiratory Symptoms (LRS)

LRS were defined as wheezing, persistent cough, or shortness of breath reported on at least one day of the 30 days preceding survey completion, excluding symptoms related to a cold, flu, or seasonal allergies [7]. LRS was measured at three time points: (1) Between 9/11 and wave 3 (prior LRS); (2) on the Hurricane Sandy survey (Sandy-related LRS); and (3) at wave 4 (persistent LRS). All three were self-reported. Prior to LRS, it was assessed as an effect measure modifier. Sandy-related LRS was examined as a primary exposure. Persistent LRS self-reported on the wave 4 survey was the primary outcome.

### 2.3. Hurricane Sandy Inhalational Exposures

We investigated three types of Hurricane Sandy-related inhalational exposures as in the previous study [5]: (1) reconstruction exposure (removing water, removing mud/debris or “muck”, “tear out” work, or major repair at the respondent’s or someone else’s homes that were damaged by Sandy); (2) exposure to mold or damp environments (for those who returned home after the hurricane, having a flooded home, seeing mold, or smelling mold at home; or being exposed to sewage, dirty or contaminated flood water, or visible mold); and (3) exposure to other respiratory irritants (i.e., debris, exhaust fumes from generators, diesel fuel or heating oil leaks, or spills). Hurricane Sandy exposures were analyzed as a count variable of the three exposure types (i.e., no exposures, one exposure, two exposures, or all three types of exposures).

### 2.4. Sandy-Related PTSD

Sandy-related PTSD was assessed on the Hurricane Sandy survey. It was measured using a version of the PTSD checklist Specific 17 (PCL-17), a 17-item self-reported symptom scale which referred specifically to the events of Sandy and corresponded to the DMV-IV criteria for PTSD (re-experiencing, avoiding, and hyper-arousal) [10,11]. Respondents reported symptoms on a five-level Likert scale ranging from “not at all” (1) to “extremely” (5), giving a possible score of 17–85. Sandy-related PTSD was defined as a Sandy survey PCL score of ≥44 [12].

### 2.5. Demographic and Physical Health Variables

Socio-demographic information (gender, age, race/ethnicity, and household income in 2010), smoking history, body mass index, and self-reported history of clinician-diagnosed asthma were obtained from Registry survey data, and all the socio-demographic data were categorized in a manner consistent with a previous study of the health effects of Hurricane Sandy [5].

### 2.6. Data Analysis

Chi-square tests were used to test the statistical significance of associations between LRS and socio-demographic characteristics, PTSD, and Hurricane Sandy exposure. Because the prevalence of LRS at wave 4 is in excess of 10%, the log binomial model with copy method [13], adjusted for variables that were associated with LRS in the previous study [5] and were significant at the bivariate level, was used to assess the risk ratios for association between the impact of Hurricane Sandy and wave 4 LRS (persistent LRS). To examine whether prior LRS modified the association between Hurricane Sandy exposures and persistent LRS, we also conducted a multivariable analysis stratified on the presence or absence of LRS before Hurricane Sandy. All analyses were conducted using SAS Version 9.4 (SAS, Cary, NC, USA).

## 3. Results

Table 1 presents sociodemographic and other select characteristics of the study sample stratified by LRS at wave 4. Participants were predominantly male (57.6%), of 45–64 years of age at the time Sandy occurred (65.9%), non-Hispanic White (73.3%), and had a household income of USD 75–150 k (38.5%). Over half had never smoked (54.4%). More than 7% reported Sandy-related PTSD, and 58.0% reported ever experiencing LRS at waves 2 or 3. Over a quarter (22.7%) reported a history of clinician-diagnosed asthma.

Over one-third (33.9%) of the sample reported at least one LRS on the wave 4 survey. Among them, 46.0% reported one LRS symptom, 26.4% reported two LRS symptoms, and 13.6% reported three LRS symptoms. Participants of an older age, male gender, lower household income, current smokers, and those with a previous asthma diagnosis were associated with at least one LRS at wave 4 in bivariate analysis. Compared to those without Sandy-related PTSD, a higher proportion of those with Sandy-related PTSD reported persistent LRS (61.8% vs. 31.7%). Moreover, a higher proportion of those who reported previous LRS also reported persistent LRS on the wave 4 survey (50.1%) compared with those who did not report previous LRS (13.1%).

Table 2 shows the association between Sandy-related predictors and persistent LRS at wave 4 in three separate multivariable models. Participants with one inhalation exposure had an adjusted risk ratio (aRR) for LRS of 1.1 (95% confidence interval [CI]: 0.98–1.25) compared with those with zero exposures. In fully adjusted models, those with two Sandy-related inhalation exposures had 1.2 times the risk of LRS (95% CI: 1.06–1.37), and those who reported three inhalation exposures were 1.19 times more likely to report LRS (95% CI: 1.07–1.33) compared with those without any exposures. In separate models, participants with Sandy-related PTSD experienced a risk of LRS aRR = 1.27 (95% CI: 1.15–1.4) compared to those without Sandy-related PTSD. Those who reported Sandy-related LRS had 1.64 times the risk of LRS at wave 4 (95% CI: 1.48–1.81) compared to those without Sandy-related LRS.

In the models assessing wave 4 persistent LRS, we discovered that the interaction between Sandy-related LRS and prior LRS, as well as the interaction between Sandy-related PTSD and prior LRS, were significant. Therefore, Table 3 examines the relationship between Sandy measures (Sandy LRS and Sandy PTSD, respectively) and persistent LRS stratified by prior LRS status. Among those without prior LRS, participants with Sandy-related PTSD had more than twice the risk of having persistent LRS (aRR: 2.4, 95% CI: 1.41–4.08). In addition, among participants who had no prior LRS, those with Sandy-related LRS had 2.56 times the risk of persistent LRS compared to those without Sandy LRS (95% CI: 1.87–3.53). No evidence was found of effect measure modification of prior LRS in the association between levels of multiple Sandy inhalation exposure and persistent LRS.

Conversely, the association between Sandy-related exposures and persistent LRS among those with prior LRS was attenuated compared to those without prior LRS. For example, in fully adjusted models, enrollees with Sandy-related PTSD had 1.26 times the risk of LRS at wave 4 (95% CI 1.15–1.38) compared to those without Sandy-related PTSD, and those with Sandy-related LRS had 1.57 times the risk of persistent LRS (95% CI: 1.42–1.74) compared to those without Sandy-related LRS.

## 4. Discussion

This study evaluated LRS symptoms three to four years after Hurricane Sandy. We found that multiple Sandy inhalation exposures (mold/damp environment, reconstruction work, and other respiratory irritants) were associated with persistent LRS. Sandy-related PTSD and Sandy-related LRS were also significantly associated with persistent LRS in separate models. In addition, we found the interaction between Sandy-related PTSD and prior LRS and the interaction between Sandy-related LRS and previous LRS were significant. Specifically, the magnitude of Sandy-related PTSD and Sandy-related LRS risk for persistent LRS was greater among those without prior LRS than those with prior LRS.

The current study found that any one of three Sandy inhalation exposures (mold/damp environment, reconstruction work, and other respiratory irritants) was not statistically associated with LRS three to four years after Sandy, while multiple Sandy inhalation exposures were associated with long-term persistent LRS. A previous study found that each of the three Sandy respiratory exposures was independently associated with LRS a few months after Sandy [5]. The current study added new evidence that the impact of each Sandy-related inhalation exposures diminished after three to four years, whereas the impact of multiple exposure remained substantial and significant.

Hurricane Sandy-related PTSD and LRS more strongly impacted persistent LRS symptoms in wave 4 among those with no previous LRS symptoms. It is unclear why this association was not consistent across sub-groups. However, this finding is similar to previous reports studying mental and behavioral health outcomes in this cohort, which found that Hurricane Sandy was more likely to negatively impact future health among those who had not previously reported psychological conditions. For example, Hurricane Sandy exposure was statistically significantly associated with re-experiencing 9/11 PTSD symptomology among those without PTSD symptoms in wave 3; however, no association was found among those who reported PTSD symptoms in the third survey wave [14]. Similarly, binge drinking was associated with Sandy-related PTSD only among those who had never experienced 9/11-related PTSD [12]. Mental and physical health are strongly linked and may work on similar causal pathways [15]. Specifically, the protective-stabilizing model of resilience states that prior trauma may neutralize the effect of future trauma exposure [16]. It is possible that those with a history of respiratory problems are less sensitized to future disaster-related exposures. Future studies about other factors that can reduce people’s LRS will be beneficial.

## 5. Conclusions

Exposure to multiple sources of potential respiratory irritants after Hurricane Sandy, including mold/damp environments, irritants from reconstruction, and diesel exhaust, was still associated with an increased risk of LRS three to four years after the hurricane, although the effect of individual inhalation exposures on the LRS decreased over time. In addition, Sandy-related LRS and Sandy-related PTSD had a prolonged impact on the enrollee’s health, and this effect was strongest among those without previous LRS symptoms. Our findings highlight the additional LRS risk among enrollees exposed to both Hurricane Sandy and the WTC disaster. This work suggests respiratory protection is important among those performing reconstruction and clean-up work after a natural disaster, particularly among those with previous adverse respiratory exposures.

## Figures and Tables

**Table 1:** Characteristics of survey participants and prevalence of Lower Respiratory Symptoms (LRS) after Hurricane Sandy among the World Trade Center Health Registry enrollees.

			LRS W4				
			Yes		No		
	Total	Column %	N	Row %	N	Row %	*p*-Value
Total	3277	100	1111	33.9	2166	66.1	
Sex							0.0058
Male	1888	57.6	677	35.9	1211	64.1	
Female	1389	42.4	434	31.2	955	68.8	
Age at wave 3							0.0014
25–44	627	19.1	175	27.9	452	72.1	
45–64	2158	65.9	770	35.7	1388	64.3	
65+	492	15	166	33.7	326	66.3	
Race							0.0037
Non-Hispanic White	2403	73.3	781	32.5	1622	67.5	
Non-Hispanic Black	285	8.7	108	37.9	177	62.1	
Latino	335	10.2	142	42.4	193	57.6	
Asian	181	5.5	57	31.5	124	68.5	
Other	73	2.2	23	31.5	50	68.5	
Income at wave 3							<0.0001
USD 50 K or less	715	22.7	275	38.5	440	61.5	
USD 50,001–75,000	513	16.3	203	39.6	310	60.4	
USD 75,001–150,000	1211	38.5	428	35.3	783	64.7	
More than USD 150 K	705	22.4	172	24.4	533	75.6	
Smoking history at wave 3							<0.0001
Never	1756	54.4	536	30.5	1220	69.5	
Former	1167	36.2	414	35.5	753	64.5	
Current	303	9.4	142	46.9	161	53.1	
Previous asthma							<0.0001
Yes	725	22.7	358	49.4	367	50.6	
No	2475	77.3	730	29.5	1745	70.5	
# of risk factors							<0.0001
0	1606	49.0	453	28.2	1153	71.8	
1	545	16.6	191	35.0	354	65.0	
2	439	13.4	172	39.2	267	60.8	
3	687	21.0	295	42.9	392	57.1	
Sandy-related PTSD							<0.0001
Yes	246	7.8	152	61.8	94	38.2	
No	2895	92.2	917	31.7	1978	68.3	
BMI at wave 3							<0.0001
obese	921	28.6	401	43.5	520	56.5	
overweight	1272	39.6	428	33.6	844	66.4	
normal/under weight	1022	31.8	262	25.6	760	74.4	
LRS at waves 2 and 3							<0.0001
Ever	1839	58.0	922	50.1	917	49.9	
Never	1330	42.0	174	13.1	1156	86.9	

**Table 2:** Adjusted Risk Ratios (aRR) for the association between Lower Respiratory Symptoms (LRS) at wave 4 and Hurricane Sandy exposures in 2015–2016 among the World Trade Center Health Registry enrollees *.

Predictor			
LRS at Wave 4	Yes vs. No	Yes vs. No	Yes vs. No
# of Sandy Inhalation exposure			
0	ref.		
1	1.10 (0.98–1.25)		
2	1.20 (1.06–1.37)		
3	1.19 (1.07–1.33)		
Hurricane Sandy-Related PTSD			
Yes		1.27 (1.15–1.40)	
No		ref.	
Hurricane Sandy-Related LRS			
Yes			1.64 (1.48–1.81)
No			ref.

* All three models are adjusted for sex, age group, race, income at wave 3, smoking history at wave 3, previous asthma, and BMI at wave 3.

**Table 3:** Adjusted Risk Ratios (aRR) for the association between Lower Respiratory Symptoms (LRS) at wave 4 and Hurricane Sandy exposures, stratified by presence/absence of prior LRS, among the World Trade Center Health Registry enrollees *.

Prior LRS	Never		Ever	
LRS.W4	Yes vs. No	Yes vs. No	Yes vs. No	Yes vs. No
Hurricane Sandy-Related PTSD				
Yes	2.40 (1.41–4.08)		1.26 (1.15–1.38)	
No	ref.		ref.	
Hurricane Sandy-Related LRS				
Yes		2.56 (1.87–3.53)		1.57 (1.42–1.74)
No		ref.		ref.

* All three models are adjusted for sex, age group, race, income at wave 3, smoking history at wave 3, previous asthma, and BMI at wave 3.

## Data Availability

The data presented in this study are available on request from the corresponding author. The data are not publicly available due to subject confidentiality.
